# Supplementary material for: High Seebeck Coefficient from Screen-Printed Colloidal PbSe Nanocrystals Thin Film
Source: Materials (Basel). 2022 Dec 9;15(24):8805. doi: 10.3390/ma15248805 (PMC9781735; doi:10.3390/ma15248805)
Supplement: Supplementary file 1 [file materials-15-08805-s001.zip › materials-2027016-supplementary.pdf]

materials

MDPI

Supplementary Material

High Seebeck Coefficient from Screen-Printed Colloidal PbSe Nanocrystals Thin Film

Viviana Sousa [1,2], Guillaume Savelli [3], Oleg I. Lebedev [4], Kirill Kovnir [5,6], José H. Correia [7,8], Eliana M. F. Vieira [7,8], Pedro Alpuim [1,2] and Yury V. Kolen'ko [2,*]

1. Center of Physics of the Universities of Minho and Porto, University of Minho, 4710-057 Braga, Portugal
2. International Iberian Nanotechnology Laboratory, 4715-330 Braga, Portugal
3. University Grenoble Alpes, CEA-Liten, 17 av. Des Martyrs, 38000 Grenoble, France
4. Laboratoire CRISMAT, UMR 6508, CNRS-ENSICAEN, 14050 Caen, France
5. Department of Chemistry, Iowa State University, Ames, IA 50011, USA
6. Ames National Laboratory, U.S. Department of Energy, Ames, IA 50011, USA
7. CMEMS-UMinho, University of Minho, 4800-058 Guimarães, Portugal
8. LABBELS–Associate Laboratory, 4710-057 Braga, Portugal

* Correspondence: yury.kolenko@inl.int

Thermoelectric characterization

To obtain the Seebeck coefficient and electrical resistivity from room temperature to 200 °C the PbSe quantum dots (QDs) thin films were measured by ZEM-3 system (ULVAC). The PbSe QD thin films deposited on substrates with size of 0.7 × 2 cm were held vertically in place among cold and heating blocks. Next, the PbSe QD thin films were heated and held at a defined temperature (30 °C, 90 °C, 140 °C, and 200 °C). After, a thermal gradient of 10 °C, 20 °C, and 30 °C was applied by heating the lower block. By measuring the temperatures from thermocouples in the centre of PbSe QD thin films and the electromotive force between the same two wires Seebeck coefficient was obtained. To determine electrical resistivity the four terminal method was applied. In this way, a current was applied at the PbSe QD thin films terminals and the voltage difference was acquired between the side wires of the thermocouple.

The custom-made equipment for measuring Seebeck coefficient at room temperature with two-probes consisted in connecting one side of the PbSe QD thin film to a heated metal block at a fixed temperature and the other side to a heat sink at room temperature, generating a temperature gradient of a few kelvin along the film. The metal block was heated by the application of successive voltage values, between 1 V and 3 V. After, the corresponding thermovoltage was measured at the PbSe QD thin film terminals. Seebeck values and errors are obtained using the LINEST function in EXCEL program. This function uses the least squares method to calculate the statistics for a straight line (that best fits the experimental data) and returns an array of parameters describing that line, including S (from the slope of the linear fitting), and error. A 7651 programmable DC source (YOKOGAWA) and a 34410A 61/2 digit multimeter (Agilent) were used for Seebeck coefficient measurements.

A scheme of the four-probe method (ZEM-3) and the two-probe custom-made equipment are shown in Figure S1.

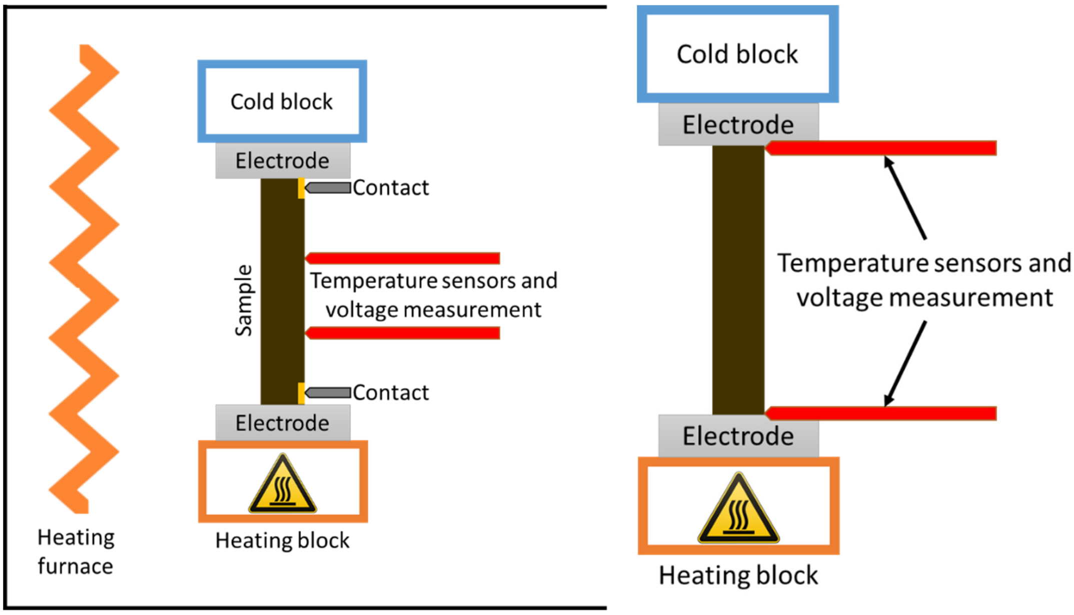

Figure S1. Scheme of four-probe method (ZEM-3) (on the left) and two-probe custom-made equipment (on the right) that were used for electrical resistivity and Seebeck coefficient measurements.

Thermogravimetric analysis

The thermal behaviour of the as-synthesized PbSe QDs was investigated by means of thermogravimetric analysis (TGA) using a TGA/DSC 1 STAR^e system (Mettler-Toledo). The powdered QDs were heated from 30 to 700 °C at 5 °C/min under a continuous Ar flow of 50 mL/min.

Thermogravimetric analysis of PbSe QDs (Figure S2) revealed that they are stable until nearly 650 °C. From room temperature to 250 °C, the observed weight loss could be related to the evaporation of the residual solvent, while detected weight loss from 250 °C and 450 °C is related to the decomposition of organic capping agent.

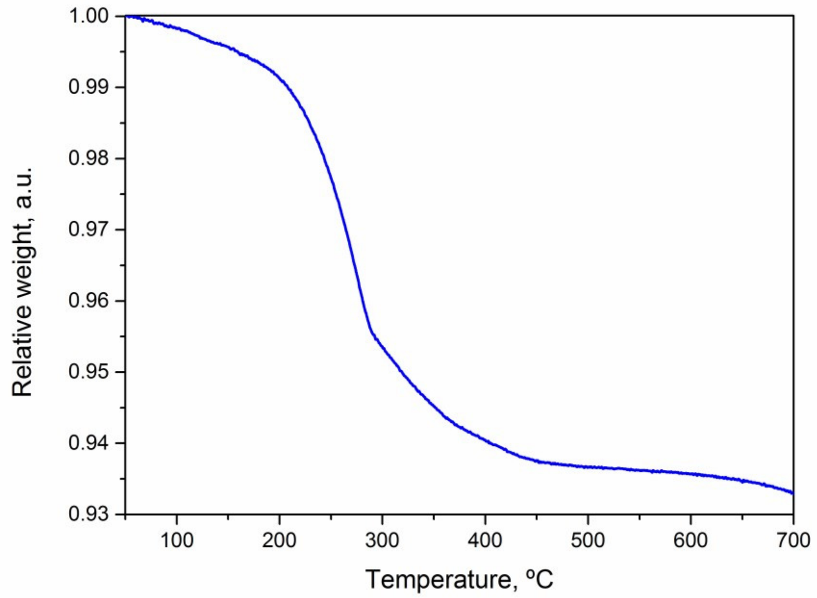

Figure S2. Thermogravimetric analysis of PbSe QDs. .

Raman spectroscopy

To inspect the presence of carbon residues in the PbSe QD thin films fabricated by screen-printing followed by heat treatment, Raman spectroscopy was performed on an alpha300 R confocal Raman microscope (WITec) using a 532 nm Nd:YAG laser for excitation. The laser beam with power of 2 mW was focused on the PbSe QD thin film by a ×50 lens (Zeiss). Afterward, the spectra was collected with an 1800 groove mm^{-1} grating using 100 acquisitions with a 2 s acquisition time.

As seen in the Figure S3, three peaks were detected by Raman spectroscopy, namely, *LO* band at 136 cm^{-1}, 2 *LO* band at 276 cm^{-1} and, Polaron at 790 cm^{-1}, which are characteristic of PbSe oxidation due to laser exposure under air atmosphere during the analysis [1,2]. The peak observed at 1350 cm^{-1} is characteristic of D_1 band related to the in-plane imperfections of graphite, such as defects and heteroatoms. The detected *G* band near 1590 cm^{-1} refers to an ideal graphite lattice vibration mode. So both bands are characteristic of carbon materials [3], suggesting the presence of the residual carbon in the resultant PbSe QD thin films.

To analyse if PbSe QD thin films were stable during thermoelectric measurements under atmospheric conditions, the as-fabricated PbSe QD thin films were subjected to a calcination at 200 °C for 30 min, and then analysed by Raman spectroscopy and XRD. No differences between as-fabricated and calcined PbSe QD thin films were observed on Raman spectra (Figure S3) or XRD pattern (Figure S4), confirming thermal stability of the films during TE properties measurements.

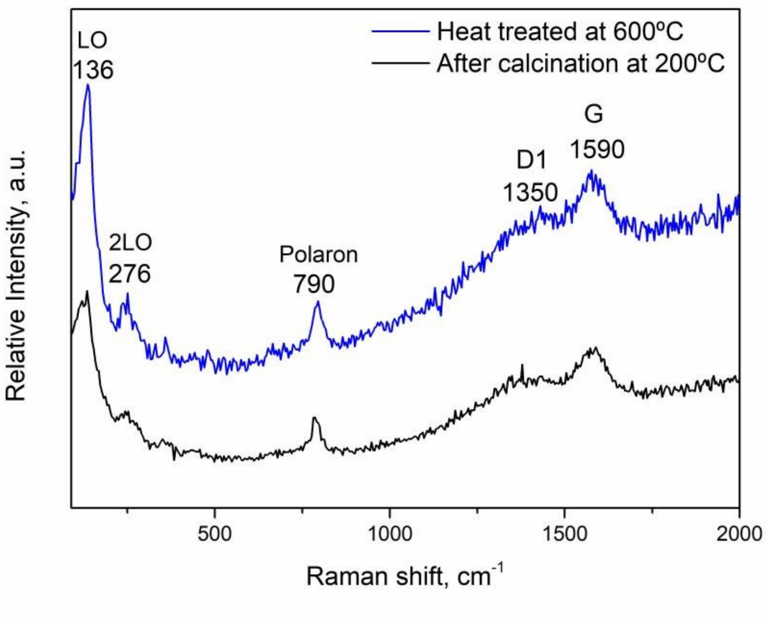

Figure S3. Raman spectra of PbSe QD thin film fabricated by screen printing followed by heat treatment and the same film after calcination at 200 °C for 30 min. .

X-ray diffraction analysis

For XRD analysis of the stability of PbSe QD film material, the calcined (200 °C, 30 min) thin film was scratched from SiO_2/Si substrate and the powder was collected and characterized. According to the XRD analysis (Figure S4), after calcination, the PbSe QDs crystalline structure is maintained and no extra peaks related with PbO appeared, which

confirms the thin film stability under atmospheric conditions during TE properties measurements up to 200 °C.

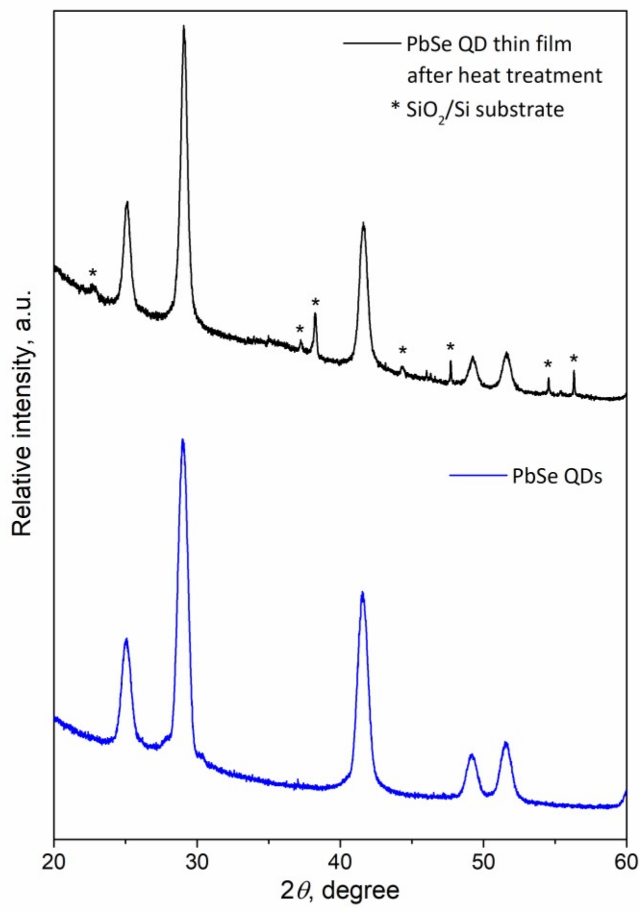

Figure S4. Comparison of the XRD patterns of the as-synthesized PbSe QDs and PbSe QD thin-film material after calcination at 200 °C for 30 min.

Thermoelectric properties

5.1. Measurement of thin films electrical properties with different methods

In the four-probe method (ZEM-3), to apply temperature difference the lower block is heated to a specific temperature and two probes near the center of the samples will measure temperature. For electrical resistivity a current is applied at sample terminals. For the two-probe custom-made equipment (2-probe), the temperature difference is applied and measured at the sample terminals.

Further analysis of different methods to measure electrical conductivity and Seebeck coefficient on PbSe QD thin films at room temperature were applied and compared in the Table S1.

Table S1. Electrical conductivity and Seebeck coefficient results for PbSe QD thin films, measured at room temperature, by different methods.

Measurement method	Hall effect	ZEM-3	2-probe
**Electrical conductivity (S m^{-1})**	50.00	28.07	88.40
**Seebeck coefficient (μV K^{-1})**	–	176.00	67.87

As presented in the Table S1, each measuring technique for determining the σ of the PbSe QD thin films led to different values at RT: σ = 50 S m^{-1} by Hall effect, σ = 28 S m^{-1} by

four-probe method, and $\sigma = 88.40$ S m^{-1} by two-probe method. Also, for the S at RT, different values were obtained from fourprobe and two-probe methods: $S = 176$ μV K^{-1} and $S = 67.87$ μV K^{-1}, respectively.

According to the literature, the value variation between four-probe and two-probe techniques is usually around ± 5% for Seebeck measurements at RT [4]. Nevertheless, for samples with high resistivity the S can be very challenging to measure accurately. During Seebeck measurement a load is applied to the sample leading to a considerable voltage drop on the internal resistance of the sample, which contributes significantly to error on Seebeck voltage measurement [5]. For instance, when S of TE bulk sample of $Co_{0.97}Ni_{0.03}Sb_3$ was measured at RT by eight different research laboratories, it varied from –140 μV K^{-1} to –210 μV K^{-1} [6]. Additionally, the surface roughness of our screen-printed 2 μm thick PbSe QD thin films could also influence contact between the film surface and the probe. To overcome the surface roughness, further improvements on ink formulation, such as PbSe QDs milling with dispersant agent and decreased content of the EC binder, could be investigated.

Thin film thickness variation

To determine if layer thickness would influence thermoelectric properties of the PbSe QD thin films, electrical conductivity and Seebeck coefficient measurements were conducted for the films with different thickness. The results obtained after measuring the films five times using two-probe custom-made equipment are presented in Table S2.

Table S2. Electrical conductivity and Seebeck coefficient results for PbSe QD thin films with different thicknesses, measured by two-probe custom-made equipment at room temperature.

**PbSe QD thin film thickness**	2 μm	3.5 μm
**Electrical conductivity (S m^{-1})**	88.40 ± 0.5	164.60 ± 6.5
**Seebeck coefficient (μ V K^{-1})**	67.87 ± 2.22	54.18 ± 2.32

References

1. Blackburn, J.L.; Chappell, H.; Luther, J.M.; Nozik, A.J.; Johnson, J.C. Correlation between Photooxidation and the Appearance of Raman Scattering Bands in Lead Chalcogenide Quantum Dots. *J. Phys. Chem. Lett.* **2011**, *2*, 599–603, doi:10.1021/jz2000326.
2. Kuzivanov, M.O.; Zimin, S.P.; Fedorov, A. V.; Baranov, A. V. Raman Scattering in Lead Selenide Films at a Low Excitation Level. *Opt. Spectrosc.* **2015**, *119*, 938–942, doi:10.1134/S0030400X15120140.
3. Liu, Y.; Sun, B.; Tajcmanova, L.; Liu, C.; Wu, J. Effect of Carbon Residues Structures on Burnout Characteristic by FTIR and Raman Spectroscopy. *Spectrochim. Acta A Mol. Biomol. Spectrosc. SPECTROCHIM ACTA A* **2022**, *272*, 120947, doi:10.1016/j.saa.2022.120947.
4. Vaqueiro, P. Chapter 1. Synthesis and Property Measurements of Thermoelectric Materials; 2021; ISBN 9781788019590.
5. Masoumi, S.; Noori, A.; Shokrani, M.; Hossein-Babaei, F. Apparatus for Seebeck Coefficient Measurements on High-Resistance Bulk and Thin-Film Samples. *IEEE Trans Instrum Meas* **2020**, *69*, 3070–3077, doi:10.1109/TIM.2019.2931066.
6. Alleno, E.; Bérardan, D.; Byl, C.; Candolfi, C.; Daou, R.; Decourt, R.; Guilmeau, E.; Hébert, S.; Hejtmanek, J.; Lenoir, B.; et al. Invited Article: A Round Robin Test of the Uncertainty on the Measurement of the Thermoelectric Dimensionless Figure of Merit of $Co_{0.97}Ni_{0.03}Sb_3$. *Rev. Sci. Instrum.* **2015**, *86*, 011301, doi:10.1063/1.4905250.
